# Phosphate Assay Kit in One Cell for Electrochemical Detection of Intracellular Phosphate Ions at Single Cells

**DOI:** 10.3389/fchem.2019.00360

**Published:** 2019-05-24

**Authors:** Haiyan Xu, Dandan Yang, Dechen Jiang, Hong-Yuan Chen

**Affiliations:** State Key Laboratory of Analytical Chemistry for Life Science, School of Chemistry and Chemical Engineering, Nanjing University, Nanjing, China

**Keywords:** nanocapillary, phosphate ions, single cells, electrochemistry, phosphate assay kit

## Abstract

In this paper, phosphate assay kit in one cell is realized for the electrochemical detection of intracellular phosphate ions at single cells. The components of the phosphate assay kit, including maltose phosphorylase, maltose, mutarotase, and glucose oxidase, are electrochemically injected into a living cell through a nanometer-sized capillary with the ring electrode at the tip. These components react with phosphate ions inside the cell to generate hydrogen peroxide that is electrochemically oxidized at the ring electrode for the qualification of intracellular phosphate ions. An average 1.7 nA charge was collected from eight individual cells, suggesting an intracellular phosphate concentration of 2.1 mM. The establishment in the electrochemical measurement of phosphate ions provides a special strategy to monitor the fluctuation of intracellular phosphate at single cells, which is significant for the future investigation of phosphate signal transduction pathway.

## Introduction

Inorganic phosphate ions are the most abundant anions inside the cells that are essential for nucleic acid and phospholipid biosynthesis, as well as for energy metabolism (Bevington et al., 1992). The concentration of intracellular phosphate is tightly maintained by membrane transporters, and the disorder of intracellular phosphate level is known to be related with multiple pathological conditions (Bun-Ya et al., 1991). For example, elevated phosphate is reported to be associated with ischemia and hypoxia, while less phosphate is related with skeletal muscle fatigue and hypophosphatemia (Bergwitz and Jüppner, 2011). Therefore, monitoring the fluctuation of intracellular phosphate is important for the biological study of phosphate signal transduction pathways. Considering the significant cellular heterogeneity, the ability to measure the phosphate level at single cells will provide more elegant information to elucidate these pathways (Wang and Bodovitz, 2010).

Many techniques, such as the colorimetry (Dickman and Bray, 1940; Cogan et al., 1999), ion chromatography (Galceran et al., 1993), fluorescence (Huang et al., 2008; Saeed et al., 2010), electrochemistry (Forano et al., 2018), and flow injection analysis (Pérez-Ruiz et al., 2001), have been well developed for the measurement of phosphate ions in the solution. The detection limit using these classical instrumental methods in the laboratories is between 20 and 150 nM (Lawal and Adeloju, 2013). To measure phosphate ions in the biological samples, a series of chemosensors were synthesized that are capable of detecting inorganic phosphates at a physiological pH (Hatai et al., 2012; Liu et al., 2016). However, very few fluorescent probes have been synthesized to recognize phosphate ions inside the cells, which are partially due to the poor selectivity for phosphate in presence of structurally similar anions in cells (Guo et al., 2015; Zhang et al., 2018).

The phosphate assay kit includes the biologically specific maltose phosphorylase that converts maltose (in the presence of phosphate) into glucose 1-phosphate and glucose. Then, glucose oxidase included in the kit reacts with glucose to generate gluconolactone and hydrogen peroxide for the colorimetric or fluorescent determination of phosphate (McDermott et al., 2006). Due to good selectivity of the enzymes in the assay, this kit has been widely applied for the specific analysis of phosphate ions in the solution (Mousty et al., 2001) and cellular lysate (Zhang et al., 2015). Accordingly, the loading of these kit components into one cell to initialize the reaction with intracellular phosphate and the immediate detection of the product (e.g., hydrogen peroxide) should provide an alternative strategy to detect phosphate ions at single cells.

Nanoelectrochemistry is a robust tool for the detection of intracellular species at single cells that positions a nanometer-sized electrode into a living cell and collects the current or charge after the electrochemical conversion of the target molecules at the electrode surface (Wightman, 2006; Wang et al., 2012; Li et al., 2014). Previously, our group designs a nanometer-sized capillary with a ring electrode at the tip that is filled with the kit components. Upon the position of the capillary inside one living cell, a voltage is applied at a metal wire in the capillary to induce electroosmotic flow (EOF) that results in the electrochemical pumping of these components into the cell (Pan et al., 2016). After the reaction between the kit components and the target molecule for a certain time, the hydrogen peroxide generated could be electrochemically detected at the ring electrode. As a result, intracellular glucose and the activity of sphingomyelinase have been successfully quantified without the significant interruption of cellular activity. Using this approach, the loading of the phosphate assay kit in one cell is feasible, and thus, the detection of phosphate ions at single cells could be realized. As compared with the previously reported approach using the synthesized probes, this kit-based method uses the commercially available kit components that avoid the complicated structural design of the probe and guarantee the specific recognition of phosphate ions.

**Figure 1:**
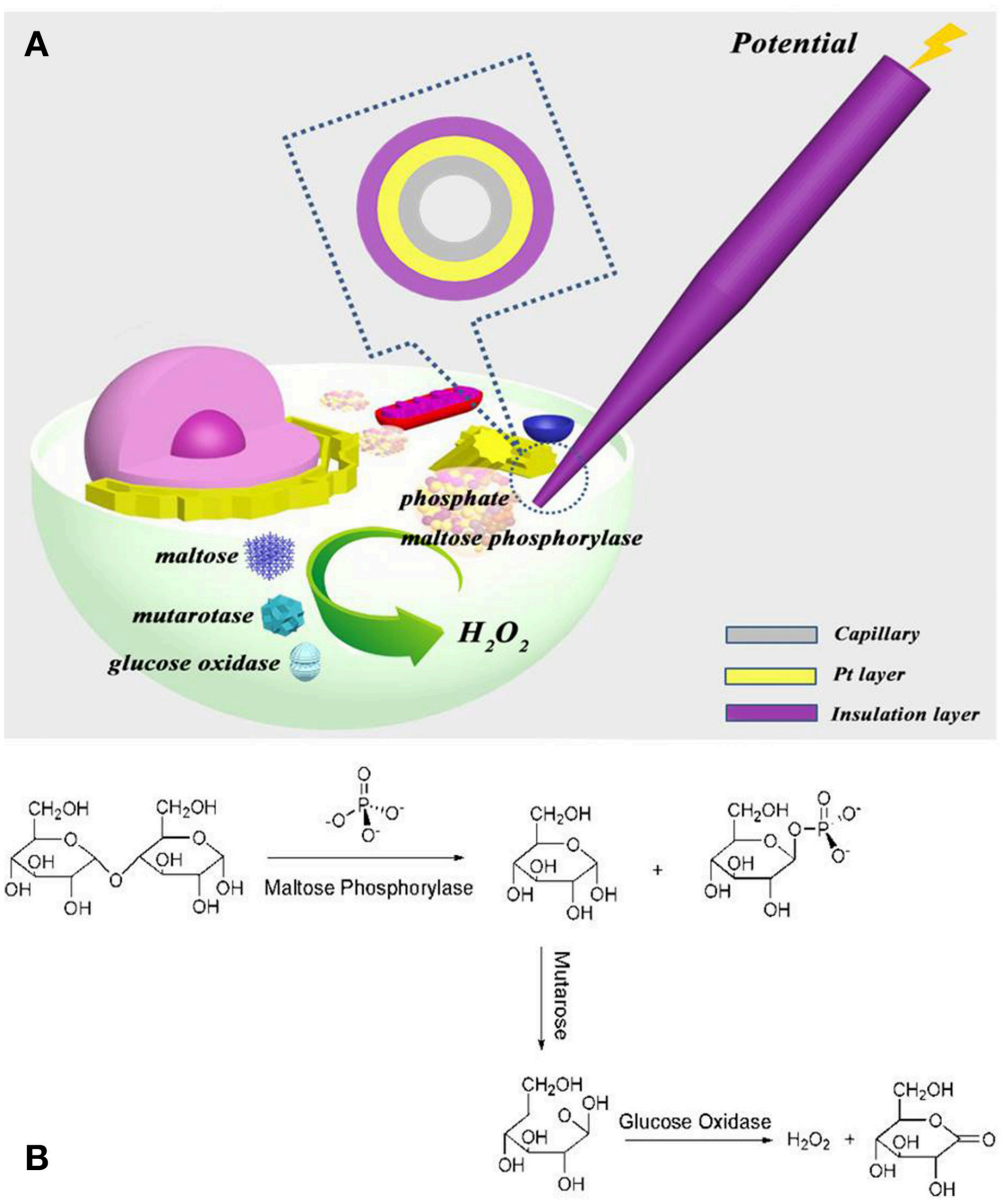
**(A)** Schematic illustration to show the electrochemical loading of the phosphate assay kit in one cell and the following electrochemical detection of intracellular phosphate ions at single cells; **(B)** the reaction of kit components (maltose phosphorylase, maltose, mutarotase, and glucose oxidase) with phosphate ions.

In this paper, the components of the phosphate assay kit, including maltose phosphorylase, maltose, mutarotase, and glucose oxidase, are filled into the nanocapillary, which is electrochemically loaded into the cell, as demonstrated in Figure 1A. Intracellular phosphate reacts with maltose phosphorylase and maltose to form α-glucose, which is transformed into β-glucose for the following oxidation by glucose oxidase (Figure 1B). The hydrogen peroxide generated is then electrochemically oxidized at the ring electrode for the quantification of phosphate ions inside the cells. The detection ability of this approach for phosphate ions in aqueous solution and inside the cells is investigated.

## Experimental Section

### Chemical and Cells

The phosphate assay kit (P22061) is obtained from Molecular Probes, Inc. (OR, USA). All the other chemicals are purchased from Sigma Chemical Co. (MO, USA). HeLa cells were obtained from the Institute of Biochemistry and Cell Biology, Shanghai Institute for Biological Science of Chinese Academy of Science (Shanghai, China). HeLa cells were cultured in DMEM/high glucose medium supplemented with 10% fetal bovine serum and 1% penicillin/streptomycin at 37°C under a humidified atmosphere containing 5% CO_2_.

### Apparatus and Measurement

The nanocapillaries with a Pt layer at the tip opening (~130 nm in diameter) are fabricated following our previously reported protocol (Pan et al., 2016). The kit components, including maltose phosphorylase (50 U/ml), maltose (40 mM), glucose oxidase (10 mg/ml), and mutarotase (50 U/ml), are filled into the capillary. A Pt wire is inserted into the capillary that is applied with a voltage of 1 V to electrochemically pump the kit components outside the capillary. Immediately after the egression and the following reaction with phosphate ions, a voltage of 0.6 V is applied at the Pt layer at the nanocapillary for the electrochemical detection using a CHI 660E electrochemical station (CH Instruments) at room temperature.

### Single-Cell Analysis

Single-cell analysis is performed under a microscope (Nikon ECLIPSE Ti-U, Nikon, Japan). Prior to each measurement, the cells are washed and re-cultured in extracellular buffer (ECB buffer: 135 mM NaCl, 5 mM KCl, 10 mM HEPES, 2 mM MgCl_2_, and 2 mM CaCl_2_) in the absence of extracellular phosphate ions. The nanocapillary is mounted on a 3D translation stage to achieve the penetration into the target cell.

## Results and Discussion

### Analysis of Phosphate Ions in the Buffer

**Figure 2:**
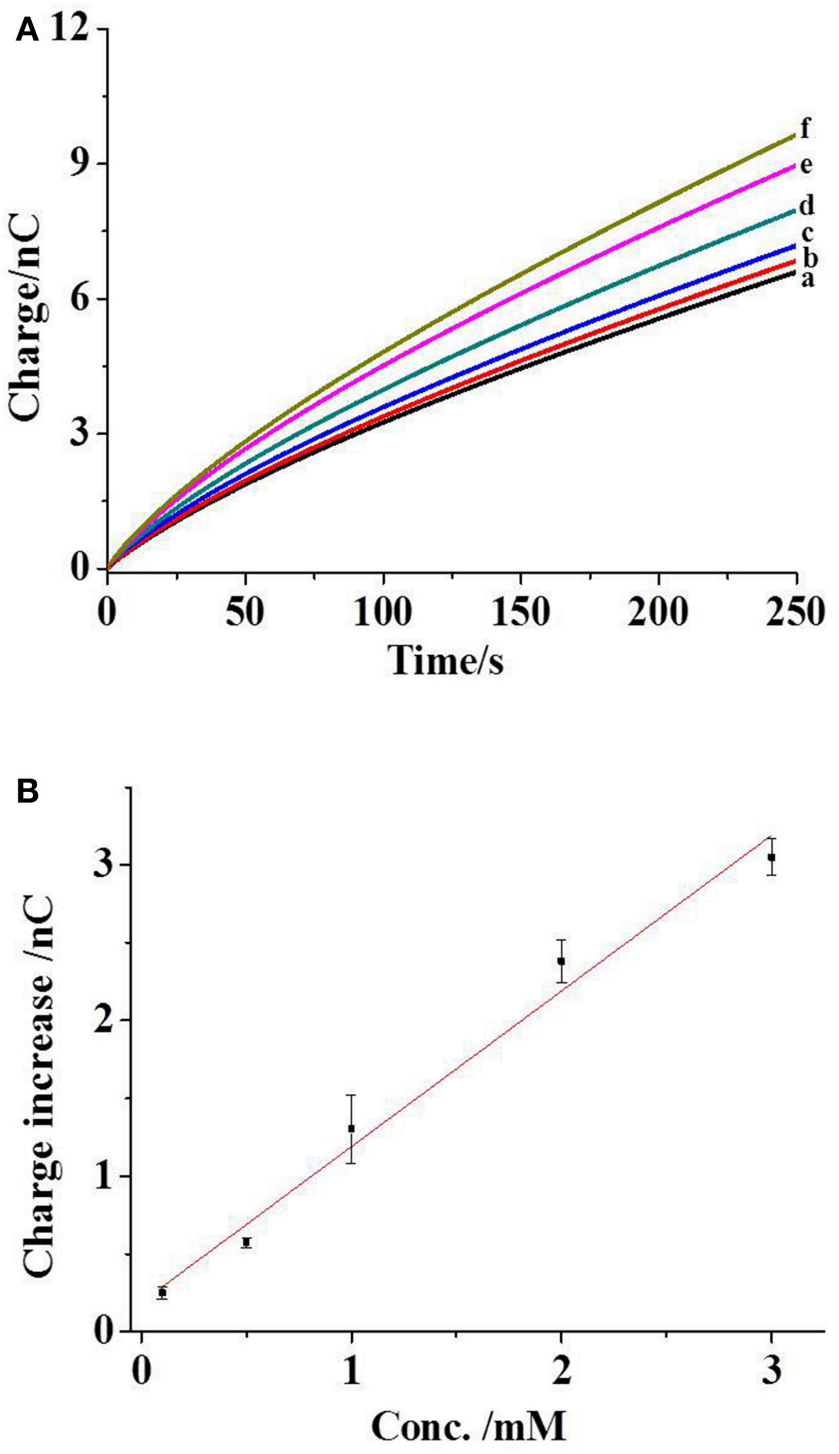
**(A)** The charges of capillary exposed to the ECB buffer (curve a), with 0.1 (curve b), 0.5 (curve c), 1 (curve d), 2 (curve e), and 3 mM phosphate ions (curve f). The capillary is filled with maltose phosphorylase (50 U/ml), maltose (40 mM), glucose oxidase (10 mg/ml), and mutarotase (50 U/ml). The condition for the electrochemical loading is 1 V for 30 s. **(B)** The correlation between the charge increase and the concentration of phosphate ions. The error bars present the standard deviations from three independent measurements. The line is the linear fitting curve.

To validate our approach for the measurement of phosphate ions, the initial experiment is conducted in ECB buffer with different concentrations of phosphate ions. As compared with 1 × phosphate buffer saline (PBS), ECB buffer has almost the same ion strength and viscosity, resulting in similar EOF rate in the capillary. Therefore, the electrochemical loading condition optimized in the previous work, including a voltage of 1 V and an application time of 30 s, is used to egress femtoliter (fl) volume of kit components outside the capillary (Pan et al., 2016). The non-faradic (or charging) charge from the Pt layer at the tip of the capillary is collected in the buffer without any phosphate ions after the electrochemical egression (Figure 2A, trace a). Once 0.1 mM phosphate ions are added into the buffer, an increase in the charge is observed (trace b) after the egression of fresh kit components. When one kit component (maltose phosphorylase, maltose, or glucose oxidase) is removed from the mixed solution inside the capillary, this charge increase disappears. Both of the results suggest the occurrence of chemical reactions of kit components and phosphate ions outside the tip that generates hydrogen peroxide and the following charge increase.

The addition of more phosphate ions in the buffer elevates the concentration from 0.1 to 3 mM. Consequently, the gradual increases in the charge are observed (Figure 2A, traces c–f), which are correlated with the concentration of phosphate ions. After subtracting the background charge, the charge increases at the time of 250 s from three independent experiments are measured and plotted with the concentrations of phosphate ions, as shown in Figure 2B. The coefficient of determination in the fitting curve is 0.98, exhibiting a near-linear relationship between the charge increase and the concentrations of phosphate ions. The relative standard deviation in this detection range is less than 16.8%. Accordingly, the charge collected in our approach could be applied to quantify the phosphate ions.

To characterize the conversion of phosphate ions by the kit components, these charge increases are compared with those collected from the measurement of glucose. Experimentally, the nanocapillaries loaded with glucose oxidase are applied to collect the charge increases in the presence of aqueous glucose, as shown in Figure S1 (supporting information). These charge increases are rationed with those from the measurement of phosphate ions, which are in the range of 20 and 56%. These values exhibit the limited conversion of phosphate ions by multiple enzymes, and thus, the control of the egression and the following detection time are critical to obtain the reproducible result. For the specificity of our measurement, various phosphate-containing species, such as pyrophosphate, hexametaphosphate, tripolyphosphate, and ATP, are measured successively using the nanocapillaries (Wang et al., 2018). As shown in Figure S2 (supporting information), no significant charge increase is observed at all these species, exhibiting good specificity of our assay.

### Single-Cell Analysis

**Figure 3:**
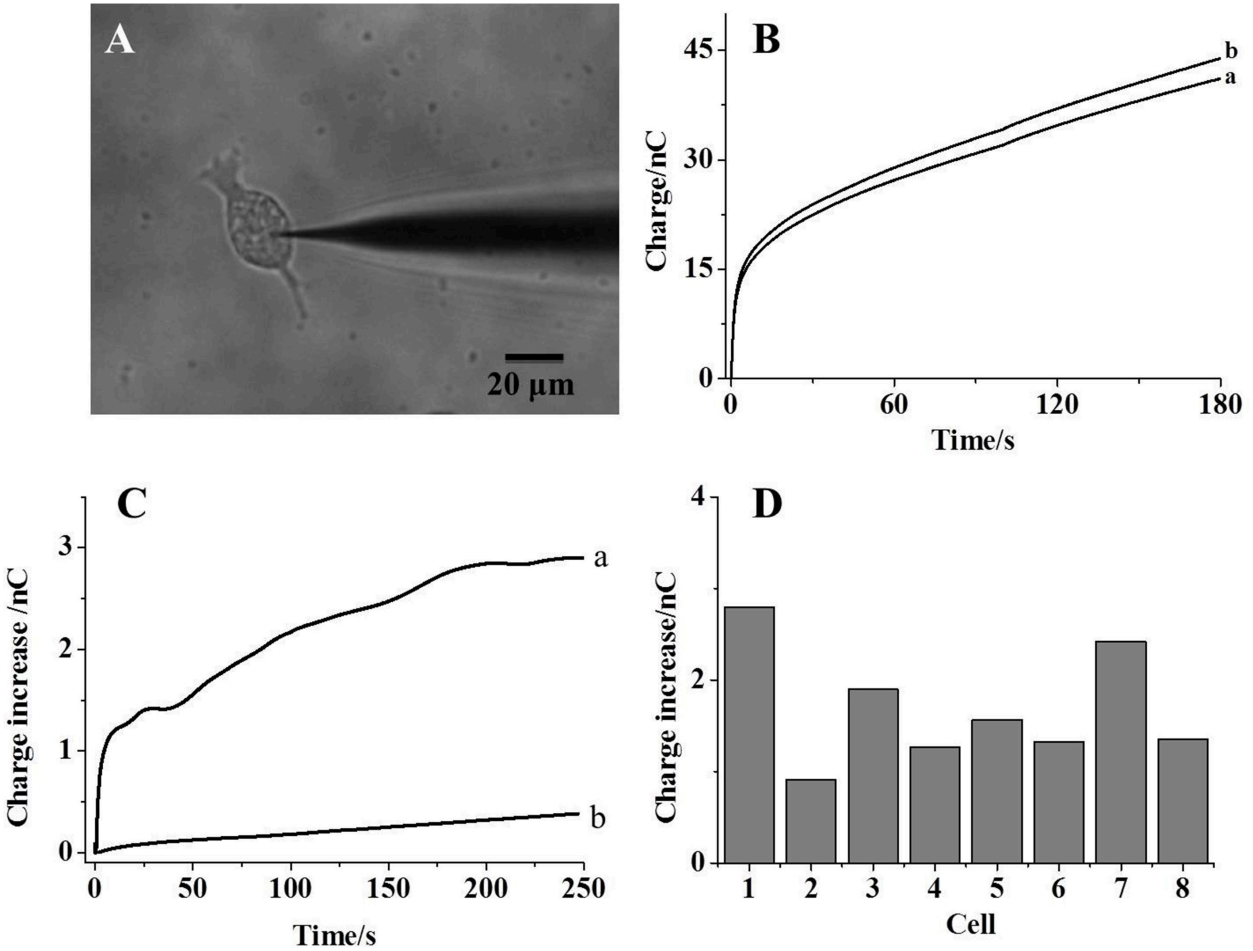
**(A)** The bright-field image of a nanocapillary inserted into a living HeLa cell; **(B)** the charges collected before (curve a) and after (curve b) the electrochemical loading of kit components into the cells; **(C)** trace a: the charge difference between curve a and b in **(B)**; trace b: the charge difference before and after the electrochemical loading of maltose, mutarotase, and glucose oxidase only into the cells; **(D)** the charge increases collected from eight individual cells.

The same measurement process is conducted after the insertion of a nanocapillary into a living cell, as imaged in Figure 3A. Because the reaction of kit components with phosphate ions generates glucose, intracellular glucose needs to be minimized to reduce the additional contribution on the charge. Therefore, the cells are starved overnight prior to each measurement, and the intracellular glucose was reported to be ~0.12 mM (Zhang et al., 2014). After the collection of the background charge (Figure 3B, trace a), a voltage of 1 V is applied at the Pt wire inside the capillary for 30 s to egress the kit components into the cell. Intracellular calcium concentration is continuously monitored using the Fluo-3 probe under the fluorescence microscope (Gobet et al., 1995). As shown in Figure S3 (supporting information), no significant increase in the calcium concentration suggests minor interruption of cellular activity during the penetration and the following electrochemical loading. After the egression of the kit components and the recording of the charge, an increase in the charge is observed (Figure 3B, trace b), which should be ascribed to hydrogen peroxide generated from the reaction with intracellular phosphate ions. To exclude the possible contribution from intracellular glucose or reactive oxygen species on the charge increase, the control experiment is performed by the introduction of the solution with glucose oxidase only. No charge increase obtained suggests that the charge increase in Figure 3B is mainly contributed by intracellular phosphate ions.

The charge difference before and after the introduction of kit components (trace a and b) is calculated and plotted in Figure 3C (trace a). An initially fast increase in the charge clearly exhibits a fast reaction between the kit components and phosphate ions. After the reaction for 200 s, the charge difference reaches the steady state, which should be attributed to the depletion of phosphate ions inside the cells. Thus, this steady-state charge increase could be used to estimate the amount of intracellular phosphate ions. Eight cells are analyzed individually and the steady-state charge increase is listed in Figure 3D. An averaged 1.7 nC is calculated from these cells with a relative standard deviation of 35.3%. According to Faraday's law, 8.5 fmol phosphate ions are detected in one cell. Because the size of a single cell is 20 μm and the cellular volume is estimated to be 4 pl, 2.1 mM phosphate ions are determined in one cell, which is consistent to the literature result (Bevington et al., 1986). Since some glucose is still present inside the starved cells, the control experiment is conducted using the nanocapillary loaded with maltose, mutarotase, and glucose oxidase only. The absence of maltose phosphorylase should not initialize the generation of glucose from phosphate ions. The charge difference before and after the introduction of these components into one cell is shown in Figure 3C (trace b). Only a slight increase in the charge supports the minor contribution of intracellular glucose on the measurement of phosphate ions inside the cells. The successful detection of intracellular phosphate ions at individual cells supports the accuracy of our approach to determine intracellular phosphate ions at single cells.

## Conclusion

In summary, the electrochemical detection of intracellular phosphate ions at single cells is realized by the loading of phosphate assay kit into one cell. This approach utilizes the commercially available kit to react with phosphate ions and, thus, avoids the design and preparation of the recognition probe. This simplification facilitates the detection of phosphate ions at single living cells. The future development will focus on the application of this approach to analyze the activity of phosphatase that releases a phosphate group from its substrate. The qualification of the activity of phosphatase, especially phosphatase and tensin homolog (PTEN), at single cells could provide the important information for the drug development. To realize this aim, the detection limit of our assay should be improved, which is undergoing in the lab.

## Author Contributions

HX and DY performed the experiments and analyzed the data. DJ and H-YC designed the project and wrote the paper.

### Conflict of Interest Statement

The authors declare that the research was conducted in the absence of any commercial or financial relationships that could be construed as a potential conflict of interest. The handling editor declared a shared affiliation, though no other collaboration, with the authors HX, DY, DJ, and H-YC at time of review.
