# Supplementary material for: Phosphate Assay Kit in One Cell for Electrochemical Detection of Intracellular Phosphate Ions at Single Cells
Source: Front Chem. 2019 May 24;7:360. doi: 10.3389/fchem.2019.00360 (PMC6542946; doi:10.3389/fchem.2019.00360)
Supplement: Supplementary file 1 [file Data_Sheet_1.docx]

Supporting information for

Phosphate Assay Kit in One Cell for Electrochemical Detection of Intracellular Phosphate Ions at Single Cells

Haiyan Xu^#^, Dandan Yang^#^, Dechen Jiang*, Hong-Yuan Chen

State Key Laboratory of Analytical Chemistry for Life Science, School of Chemistry and Chemical Engineering, Nanjing University, Nanjing 210093, China

Corresponding Author

Phone/Fax: 086-25-83594846

E-mail: [dechenjiang@nju.edu.cn](mailto:dechenjiang@nju.edu.cn)

# These authors contribute equally

Figure S1. The correlation between the charge increase and the concentration of glucose using the nano-capillaries loaded with glucose oxidase. The error bars present the standard deviations from three independent measurements.

Figure S2. The charge increases collected from the nano-capillaries in the measurement of 1 mM phosphate, pyrophosphate, hexametaphosphate, tripolyphosphate and ATP. The error bars present the standard deviations from three independent measurements.

Figure S3. The normalized fluorescence intensity (or calcium concentration) inside the fluo-3 stained cells before and after the insertion of nano-capillary into the cell and the following electrochemical pumping. The error bars present the standard deviations from three independent measurements.
